# Claudin-1, -3 and -4 proteins and mRNA expression in benign and malignant breast lesions: a research study

**DOI:** 10.1186/bcr983

**Published:** 2005-01-31

**Authors:** Anna-Mária Tőkés, Janina Kulka, Sándor Paku, Ágnes Szik, Csilla Páska, Pál Kaposi Novák, László Szilák, András Kiss, Krisztina Bögi, Zsuzsa Schaff

**Affiliations:** 1 2nd Department of Pathology, Semmelweis University, Budapest, Hungary; 2 Department of Molecular Pathology, Joint Research Organization of the Hungarian Academy of Sciences, Budapest, Hungary

**Keywords:** breast, claudin, immunohistochemistry, real-time PCR, tight junction

## Abstract

**Introduction:**

We compared levels of protein and mRNA expression of three members of the claudin (CLDN) family in malignant breast tumours and benign lesions.

**Methods:**

Altogether, 56 sections from 52 surgically resected breast specimens were analyzed for CLDN1, CLDN3 and CLDN4 expression by immunohistochemistry. mRNA was also analyzed using real-time PCR in 17 of the 52 cases.

**Results:**

CLDNs were rarely observed exclusively at tight junction structures. CLDN1 was present in the membrane of normal duct cells and in some of the cell membranes from ductal carcinoma *in situ*, and was frequently observed in eight out of nine areas of apocrine metaplasia, whereas invasive tumours were negative for CLDN1 or it was present in a scattered distribution among such tumour cells (in 36/39 malignant tumours). CLDN3 was present in 49 of the 56 sections and CLDN4 was present in all 56 tissue sections. However, CLDN4 was highly positive in normal epithelial cells and was decreased or absent in 17 out of 21 ductal carcinoma grade 1, in special types of breast carcinoma (mucinous, papillary, tubular) and in areas of apocrine metaplasia. CLDN1 mRNA was downregulated by 12-fold in the sample (tumour) group as compared with the control group using GAPDH as the reference gene. CLDN3 and CLDN4 mRNA exhibited no difference in expression between invasive tumours and surrounding tissue.

**Conclusions:**

The significant loss of CLDN1 protein in breast cancer cells suggests that CLDN1 may play a role in invasion and metastasis. The loss of CLDN4 expression in areas of apocrine metaplasia and in the majority of grade 1 invasive carcinomas also suggests a particular role for this protein in mammary glandular cell differentiation and carcinogenesis.

## Introduction

In epithelial cells, tight junctions (TJs) are the most apical intercellular junctions, and function as selective barriers to macromolecules and prevent diffusion of lipids and proteins between apical and basolateral membrane domains. TJs appear as a network of continuous and anastomosing filaments on the protoplasmic face of the plasma membrane [1]. The molecular architecture of TJ strands has been described in recent years. The proteins involved in the formation of TJs have been divided into two categories: integral membrane proteins, such as claudins (CLDNs), occludins and JAM (junctional adhesion molecule) [2]; and peripheral membrane proteins, such as zonulae occludens (ZO)-1, ZO-2 and ZO-3, cingulin, symplekin, pilt, MAGI-1 (MAGUK [membrane associated guanylate kinase] inverted protein 1) and AF-6 (afadin) [3].

CLDNs, a family comprising 24 members, are the main family of proteins that make up the TJs [2,4]. The tightness of individual TJ strands *in situ *is determined by the various combinations of CLDN family members. CLDNs have a membrane topology similar to that of occludin, even though they are smaller proteins of about 22 kDa. Their protein structure, which is uniform among family members, contains four transmembrane domains with amino- and carboxyl-termini in the cytoplasm and two extracellular loops [5].

Until now only few data have been reported regarding the role played by CLDNs in breast lesions, and findings on their function remain controversial [6,7]. There is 38% homology between human CLDN1 and CLDN2 at the amino acid sequence level [8,9]. Expression of the four-exon CLDN1 gene, formerly named senescence associated epithelial membrane protein, was identified in testis [10], colorectal carcinoma [11] and lung [12]. The direct involvement of CLDN1 in the barrier function of TJs has been demonstrated in CLDN1-over-expressing MDCK cells [13].

In a study conducted in breast carcinomas by Kramer and coworkers in 2000 [7] it was shown that there are no genetic alterations in the promoter or coding sequences in the CLDN1 gene that could account for the loss of CLDN1 protein expression in tumour cells. Of the relatively few publications concerning CLDN expression in the breast, one study [14] found that CLDN7 protein and mRNA are downregulated in primary breast cancers as compared with normal breast epithelium. Taken together, these observations indicate that there is a need to study the possible role played by CLDNs in carcinogenesis and tumour differentiation.

CLDN3 and CLDN4 can function as receptors for *Clostridium perfringens *enterotoxin [15]. The one-exon genes of CLDN3 and CLDN4 are closely linked (CLDN3 7q11 and CLDN4 7q11.23) and share extended homology of approximately 80% at the DNA level. SAGE (serial analysis of gene expression) demonstrated that, of differentially upregulated mRNAs, CLDN3 and CLDN4 were among the six most upregulated in primary ovarian carcinoma cells [16]. Van Itallie and coworkers [17] demonstrated the close correlation between the level of CLDN4 expression and change in both conductance and ionic selectivity.

Thus far there is no evidence on how CLDN3 and CLDN4 are expressed in breast carcinomas. The present study was designed to determine whether human CLDN1, CLDN3 and CLDN4 are expressed in different types of breast lesions, what differences there are between different CLDNs in breast, and how the protein expression correlates with CLDN mRNA expression.

## Methods

### Materials

For immunohistochemistry, paraffin-embedded sections were used. For immunofluorescence, paraffin-embedded sections and frozen resected breast tissue samples were analyzed. For real-time PCR RNA isolated from frozen surgical breast tissues were analyzed.

#### Materials used for immunohistochemistry

Altogether, 56 paraffin-embedded sections from 52 surgically resected breast specimens were analyzed for expression of CLDN1, CLDN3 and CLDN4 using immunohistochemistry. Tissue samples were collected with the patient's written consent and conforming with national law (23/2002.V.9 EüM) regarding human studies. The age of the 52 female patients ranged between 31 and 80 years. Carcinomas were graded according to the Elston-Bloom and Richardson system [18].

The histopathological diagnoses of the selected breast lesions were as follows: fibrocystic changes/breast lesions (*n *= 12), ductal carcinoma *in situ *(DCIS; *n *= 5), invasive ductal carcinoma (IDC) ± DCIS (*n *= 27), invasive lobular carcinoma (ILC; *n *= 5) and special types of breast carcinoma (papillary, mucinous, tubular; *n *= 7).

#### Materials used for immunofluorescene

Immunofluorescence detection of CLDN1, CLDN3 and CLDN4 was performed on frozen sections (IDC and surrounding breast) as well as on tissue microarray of IDCs obtained from paraffin-embedded blocks.

#### Materials used for CLDN1, CLDN3, CLDN4 and GAPDH mRNA expression with real-time PCR

In 17 out of the 52 cases analyzed by immunohistochemistry, the level of mRNA expression of CLDN1, CLDN3 and CLDN4 were also analyzed using real-time PCR. The histopathological diagnoses of these 17 breast lesions are as follows: fibrocystic changes/breast lesions (*n *= 3), IDC ± DCIS (*n *= 12), ILC (*n *= 1) and intraductal papilloma (*n *= 1). In the following text only the real-time PCR results for the 13 invasive carcinomas (12 IDC ± DCIS and 1 ILC) are discussed in detail.

Because relative quantification of the real-time PCR results was used for data analyses in all 17 breast samples, the normal component of the breast tissue was simultaneously investigated for mRNA expression of CLDN1, CLDN3, CLDN4 and glyceraldehyde-3-phosphate dehydrogenase (GAPDH) mRNA expression. GAPDH was used as the reference gene, and so data are normalized to GAPDH.

Breast tissue samples, obtained in accordance with current local and ethical recommendations, were snap frozen in liquid nitrogen within 30 min of surgical removal and stored at -80°C.

### Immunohistochemistry for CLDN1, CLDN3 and CLDN4 detection

Immunohistochemical detection of CLDNs was performed in formalin-fixed, paraffin-embedded sections. Sections (5 μm thick) were cut from each paraffin block, deparaffinized and rehydrated. Immunohistochemical staining for CLDN1, CLDN3 and CLDN4 was performed using the streptavidin-peroxidase procedure.

For CLDN1 and CLDN3 proteins, antigen retrieval was performed with two different antigen retrieval solutions in parallel sections: 20 min in Vector (Burlingame, CA, USA) antigen retrieval solution in a microwave oven; and 40 min in DAKO (Carpinteria, CA, USA) antigen retrieval solution in a microwave oven. For CLDN4, antigen retrieval was performed for 20 min in Vector antigen retrieval solution in a microwave oven. Endogenous peroxidase was blocked with 1% H_2_O_2 _for 10 min at room temperature.

Sections were incubated with primary antibodies (Zymed, San Francisco, CA, USA) to CLDN1, CLDN3 and CLDN4 (concentration 1:100) overnight. The antibodies used were polyclonal rabbit anti-CLDN-1 (Zymed; reactivity: human, rat and dog), polyclonal rabbit anti-CLDN-3 (Zymed; reactivity: human, mouse and dog) and monoclonal mouse anti-CLDN-4 (Zymed; reactivity: human).

Antigen-bound primary antibody was detected using standard avidin–biotin immunoperoxidase complex (DAKO LSAB2 Kit). The chromogen substrate was aminoethylcarbazol. In each case, for counter-staining Mayer's haemalaun was used. For each CLDN a negative control with omission of the primary antibody was included. Colon carcinoma was used as positive control. In each case, two independent observers (A-MT and JK) recorded the distribution of staining, intensity and localization. Staining of CLDNs was expressed relative to adjacent normal mammary epithelium.

### Immunofluorescence microscopy for CLDN1, CLDN3 and CLDN4 detection

Sections (10 μm thick) were cut from frozen tissue and from breast tissue array paraffin blocks. The tissue arrays were constructed in our laboratory. The selected breast tumour samples were obtained from paraffin blocks from our files. In brief, the study involved breast tumours from 30 T1N0 and 30 T1N1 cases. Histopathological data included tumour stage according to the UICC TNM system. With the guidance of an experienced pathologist (JK), representative core tissue biopsies (2 mm in diameter) were taken from archival paraffin-embedded primary breast carcinomas and seeded in new paraffin blocks using an instrument provided by Histopathology Company (Pécs, Hungary). Two biopsies were taken from each paraffin block. For paraffin-embedded sections antigen retrieval was performed as described above (under Immunohistochemistry for CLDN1, CLDN3 and CLDN4 detection).

Sections were incubated with goat antiserum for 1 hour at room temperature and then incubated with the primary antibodies (Zymed) to CLDN1, CLDN3 and CLDN4 (1:50) overnight. CLDN1 and CLDN3 antigen-bound primary antibody was detected using anti-rabbit IgG conjugated to Alexafluor 488 (Molecular Probes, Eugene, OR, USA) and CLDN4 stained sections was detected using anti-mouse IgG conjugated to Alexafluor 488 (Molecular Probes) for 30 min at room temperature. Cell nuclei were counter-stained with propidium iodide (Molecular Probes) for 10 min at room temperature. For each CLDN a negative control with omission of the primary antibody was included. Coverslips were mounted with fluorescent mounting medium (DAKO). Stained specimens were analyzed by laser scanning confocal microscopy (MRC 1024; Bio-Rad, Hercules, CA, USA).

### Western blotting

The monospecificity of CLDN1, CLDN3 and CLDN4 antibodies was tested by our group in whole tumour cell lysate by Western blot analyses, as described previously [14]. In brief, from snap frozen tissues of IDC of the breast and normal tissue surrounding the tumour, total protein was extracted using lysis buffer (100 mmol/l NaCl, 1% NP-40, 2 mmol/l EDTA, 50 mmol/l TRIS, 20 μg/ml aprotinin, 20 μg/ml leupeptin, 1 mmol/l PMSF, pH 7.5). Subsequently, the samples were mixed with 2× Laemmli sample buffer and boiled for 10 min, followed by centrifugation at 13,000 rpm for 15 min. Similar amounts of protein were loaded in each lane and run on 10% SDS-PAGE under reducing conditions. Then, gel electrophoresis proteins were transferred to nitrocellulose membranes. For immunodetection, blots were incubated with CLDN1 polyclonal antibody (1:400), CLDN3 polyclonal antibody (1:800) and CLDN4 monoclonal antibody (1:200) overnight. The biotinylated secondary antibody, goat anti-mouse IgG (diluted 1:2000, E433; DAKO), was applied for 1 hour at room temperature. Antibody detection was conducted using an enhanced chemiluminescent reaction system.

### Real-time PCR for CLDN1, CLDN3, CLDN4 and GAPDH mRNA detection

#### RNA isolation

Breast tissue (50–100 mg) was homogenized and RNA was isolated with Trizol (Invitrogen, Carlsbad, CA, USA) [19], in accordance with the manufacturer's instructions, using a Polytron homogenizer (Kinematica AG, Littau/Lucerne, Switzerland). Briefly, the RNA was precipitated with 0.5 ml isopropyl alcohol in the aqueous phase. The RNA pellet was washed once in 70% ethanol, dried and resuspended in 50 μl of RNAse-free water and kept at -80°C until use. Total RNA integrity was verified by electrophoresis using ethidium bromide staining as well as by measuring optical density (OD). OD 260 nm/OD 280 nm absorption ratio was accepted between 1.70 and 2.8.

#### Reverse transcription of RNA

Total RNA (700 ng) was reverse transcribed for 40 min at 48°C in 30 μl with 2.5 unit M-MuLV reverse transcriptase (Applied Biosystems, Foster City, CA, USA) in the presence of RNAse inhibitor (Applied Biosystems) using Random Hexamers (Applied Biosystems).

#### Real-time PCR

Conditions for real-time PCRs were optimized in a gradient cycler (Mastercycler Gradient, Eppendorf, Hamburg, Germany). Optimized conditions were transferred to the Bio-Rad real time PCR detection system as follows. Typical real-time PCR reaction was conducted with 2 μl cDNA template in a total volume of 25 μl, using the Bio-Rad iCycler Real Time PCR detection system (BioRad 1708740). For CLDNs and GAPDH the SYBR Green based real-time PCR method was used. Each PCR was conducted in 25 μl volume of 1 × PCR puffer (BioRad 1708882) with 300 nmol/l of each primer (Table 1) for 2 min at 95°C for initial denaturing, then 40 cycles of 95°C for 20 s, 63°C for 30 s, and 72°C for 30 s, followed by melting analyses from 55 to 95°C. Melting curve analyses resulted in single product specific melting temperatures for the analyzed CLDN1, CLDN3, CLDN4 and GAPDH genes. No primer–dimer formations were observed during the 40 real-time PCR amplification cycles. PCR reaction for each sample was done in duplicate in 96-well plates. In addition, the resulting real-time PCR product (10 μl) was loaded onto 2% agarose gel prepared in Tris-borate EDTA puffer and stained with ethidium bromide (Bio-Rad). Each PCR was optimized to ensure that no bands corresponding to genomic DNA amplification or primer–dimer pairs were present. The relative quantification method (described in detail by Pfaffl and coworkers [20] from the Technical University of Munich) was used for data analysis of real-time PCR. The program included a statistical evaluation. Data were analyzed for significant differences by analysis of variance using approximate tests [20]. The relative expression is based on the expression ratio of a target gene versus a reference gene (GAPDH).

### Electron microscopy

In two cases both the normal breast tissue and the tumour sample were investigated by transmission electron microscopy in order to analyze the presence of TJs in tumours compared with nontumourous tissue, as described previously [21].

## Results

Immunohistochemical localization of CLDNs indicated that CLDNs were expressed along the entire length of the lateral plasma membranes between epithelial cells, including apical areas containing TJ structures. Using electron microscopy, TJ structures were identified in normal breast as well as in breast tumour tissue. Cell types in breast tissue are heterogeneous, and it is therefore noteworthy that CLDNs were expressed only in epithelial cells. Surrounding fibroblasts and adipocytes served as negative controls because these cells were negative for CLDN antibodies.

### Immunohistochemistry of CLDN1

**Figure 1:**
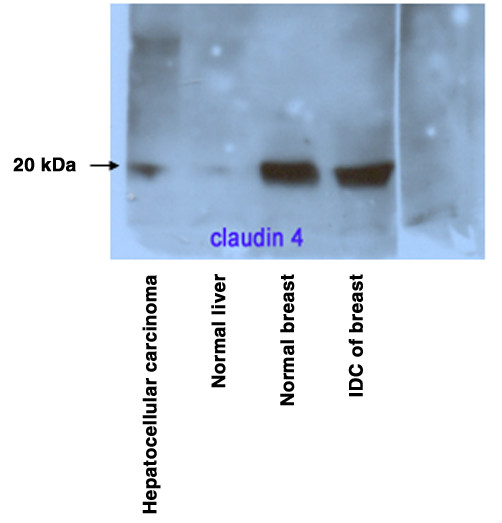
The monospecificity of claudin (CLDN)4 antibody using Western blot analysis. CLDN4 protein expression in invasive breast carcinoma, surrounding normal breast and human liver. Western blot analysis was performed on equal amounts of protein from total cell lysates using CLDN4 monoclonal antibody. A single band was shown at approximately 22 kDa for CLDN4. IDC, invasive ductal carcinoma.

The two antigen retrieval methods used yielded similar results. The monospecificity of the CLDN antibodies was tested by Western blot analyses, and a single band was found at approximately 20 kDa for CLDN1, CLDN3 and CLDN4 (Fig. 1).

**Figure 2:**
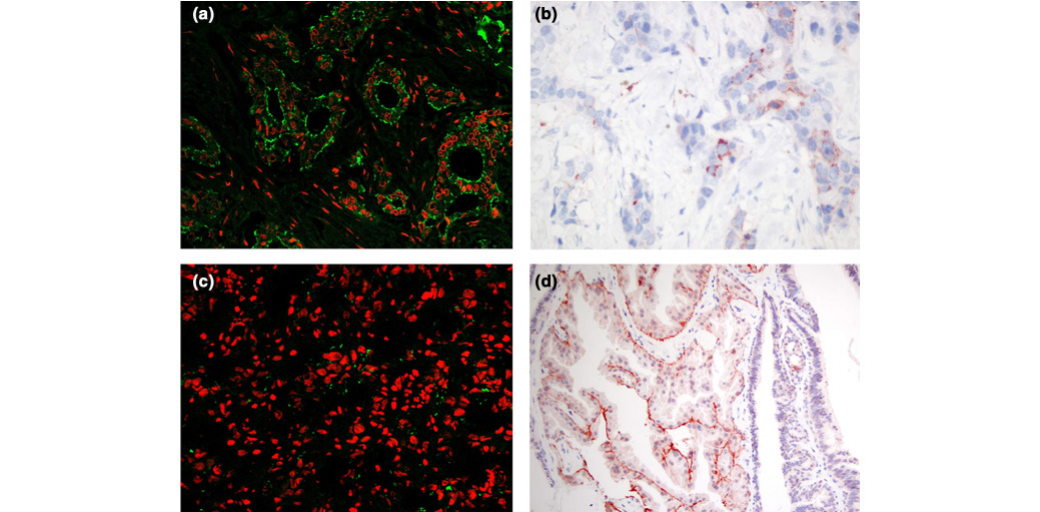
Claudin (CLDN)1 positivity in normal breast epithelium, tumour cell membranes and intraductal papilloma. **(a) **CLDN1 positivity in normal breast epithelium. Normal epithelial cells exhibit intensive CLDN1 positivity in the cell membrane. Image obtained using laser scanning confocal microscopy (MRC 1024; Bio-Rad). Primary antibody was CLDN1 polyclonal antibody (Zymed); secondary antibody was IgG conjugated to Alexafluor 488 (Molecular Probes). Cell nuclei were counter-stained with propidium iodide. Green fluorescence along the cell membranes corresponds to CLDN1; the nuclei are seen in red. (Original magnification: 400×.) **(b) **CLDN1 positivity in tumour cell membranes. Shown is the immunohistochemical reaction of invasive ductal carcinoma of the breast using polyclonal CLDN-1 antibody. Note the membrane staining only in some scattered tumour cells, as compared with normal epithelial cells (panel a). Sections were counter-stained with Mayer's haemalaun. The chromogen substrate was aminoethylcarbazol. (Original magnification: 600×.) **(c) **CLDN1 positivity in tumour cell membranes. Shown is the immunohistochemical reaction of invasive ductal carcinoma using polyclonal CLDN1 antibody. There is scattered membrane staining in some tumour cells. The secondary antibody is IgG conjugated to Alexafluor 488. Cell nuclei were counter-stained with propidium iodide. The image was obtained using laser scanning confocal microscopy (MRC 1024; Bio-Rad). (Original magnification: 400×.) **(d) **CLDN1 positivity in intraductal papilloma. Shown is the immunohistochemical reaction of a breast papilloma with an area of apocrine metaplasia. There is increased CLDN1 positivity in apocrine cells and negative staining in the surrounding papilloma. Sections were counter-stained with Mayer's hemalaun. The chromogen substrate was aminoethylcarbazol. (Original magnification: 200×.)

Immunohistochemical localization of CLDN1 indicated that CLDN1 was present in the membranes of normal duct cells in the majority of cases (Fig. 2a). CLDN1 positivity was also observed in some cell membranes in pure DCIS cases or in the DCIS component of invasive carcinomas. On the other hand, major differences in CLDN1 expression were observed among tumours.

Two major differences were noticed. First, in 36 of the 39 carcinomas examined, invasive tumour cells were either negative for CLDN1 or it was present in a scattered distribution among such tumour cells (Fig. 2b). These findings were confirmed by confocal microscopy images (Fig. 2c). There was no difference immunohistochemically in CLDN1 expression between different types of invasive carcinomas or between ductal carcinomas of different grade. The second major difference from normal tissue was that CLDN1 was frequently present (eight out of nine samples) in areas of apocrine metaplasia (Fig. 2d). In these cases positivity was observed only in cells of apocrine metaplasia, and the neighbouring tumour tissue was negative for CLDN1.

### Immunohistochemistry of CLDN3 and CLDN4

**Figure 3:**
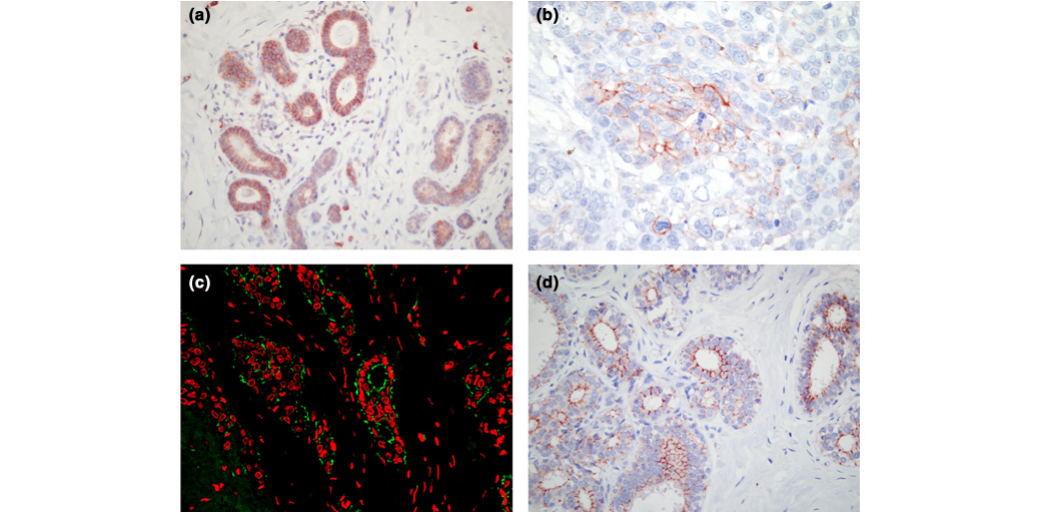
Claudin (CLDN)3 positivity in benign breast tissue and invasive ductal breast carcinoma. **(a) **CLDN3 positivity in benign breast tissue. Regular immunohistochemical reaction of benign breast epithelial cells using polyclonal CLDN3 antibody. Continous membrane staining characterizes most of the luminal epithelial cells. Sections were counter-stained with Mayer's haemalaun. The chromogen substrate was aminoethylcarbazol. (Original magnification: 400×.) **(b) **CLDN3 positivity in invasive ductal breast carcinoma. CLDN3 positivity is apparent in the membranes of some carcinoma cells. Sections were counter-stained with Mayer's hemalaun. The chromogen substrate was aminoethylcarbazol. (Original magnification: 600×.) **(c) **CLDN3 positivity in benign breast epithelium. CLDN3 positivity is seen in the majority of the membranes of benign epithelial cells. CLDN3 appeared to be localized in normal breast close to the apical end of the cell membranes or in the basolateral membranes of the epithelial cells. The primary antibody was CLDN3 polyclonal antibody (Zymed); the secondary antibody was IgG conjugated to Alexafluor 488 (Molecular Probes). Cell nuclei were counter-stained with propidium iodide. The image was obtained using laser scanning confocal microscopy (MRC 1024; Bio-Rad). (Original magnification: 400×.) **(d) **CLDN3 positivity in benign breast epithelium. CLDN3 positivity is seen in the membranes of the majority of breast epithelial cells. The positive membrane reaction is incomplete in most of the cells. The primary antibody was CLDN3 polyclonal antibody (Zymed). Sections were counter-stained with Mayer's haemalaun. The chromogen substrate was aminoethylcarbazol. (Original magnification: 400×.)

CLDN3 positivity was observed in the majority (49/56) of cases examined (12 fibrocystic breasts, 5 DCIS, 23 IDC, 4 ILC, 2 mucinous, 2 tubular and 1 papillary breast carcinomas) and in all cases analyzed by confocal microscopy. There were no remarkable differences in CLDN3 expression in different types of breast lesions or between normal and tumour components (Fig. 3a,b). CLDN3 expression is evaluable by standard immunohistochemistry and by comparing two antigen retrieval methods; in the majority of cases CLDN3, similar to CLDN1, was observed along the entire length of the epithelial cell membrane, but in a few cases, especially in normal tissues and in tissue with fibrocystic changes, CLDN3 appeared to be localized close to the apical end of the cells or at the basolateral membrane of the epithelial cells. These findings were seen on both confocal and light microscopy (Fig. 3c,d).

**Figure 4:**
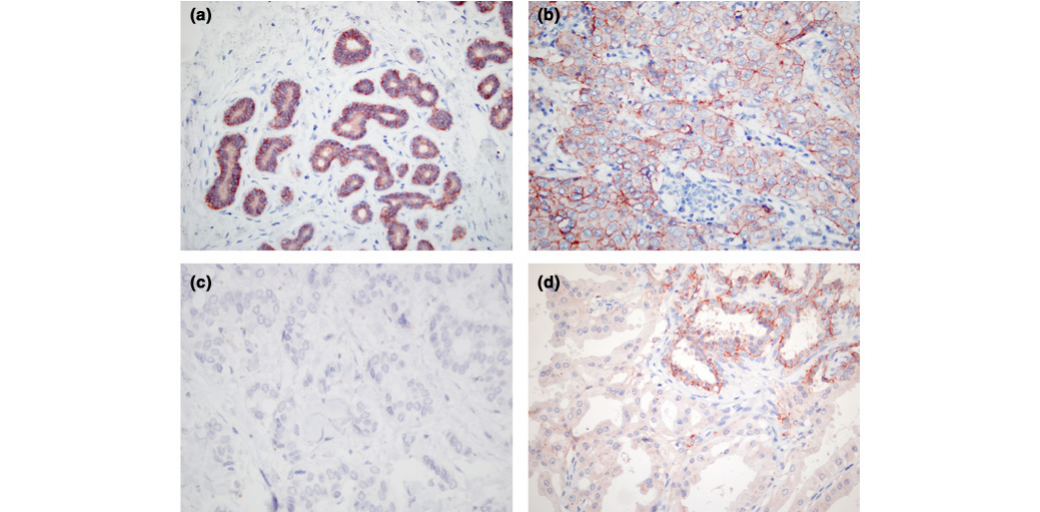
Claudin (CLDN)4 expression in benign breast epithelium, in invasive ductal carcinomas and in intraductal papilloma. **(a) **Intense CLDN4 positivity in benign breast epithelium. Shown is the immunohistochemical reaction of benign breast using monoclonal CLDN4 antibody (Zymed). Strong positivity is seen in epithelial cell membranes. Sections were counter-stained with Mayer's haemalaun. The chromogen substrate was aminoethylcarbazol. (Original magnification: 400×.) **(b) **CLDN4 positivity in invasive ductal breast carcinoma. CLDN4 is expressed in invasive ductal breast carcinoma of grade 3. Positive reaction is evident in the membranes of the tumour cells. Sections were counter-stained with Mayer's haemalaun. The chromogen substrate was aminoethylcarbazol. (Original magnification: 600×.) **(c) **CLDN4 expression in invasive ductal carcinoma of the breast. Shown is complete loss of CLDN4 expression in invasive ductal breast carcinoma of grade 1 as compared with grade 3 (panel b). Sections were counter-stained with Mayer's haemalaun. The chromogen substrate was aminoethylcarbazol. (Original magnification: 400×.) **(d) **CLDN4 positivity in intraductal papilloma. Shown is the immunohistochemical reaction of breast papilloma with an area of apocrine metaplasia. The loss of CLDN4 positivity in apocrine cells and the positive staining of other epithelial cells is a 'mirror image' of that seen in the CLDN1 reaction (Fig. 2d). Sections were counter-stained with Mayer's haemalaun. The chromogen substrate was aminoethylcarbazol. (Original magnification: 400×.)

CLDN4 positivity was present in all 56 tissue sections in epithelial cell membranes as well as in the frozen section and breast tumour array analyzed by confocal microscopy. Intense positivity was observed in normal epithelial cells (Fig. 4a) and in tumours of grade 2 and grade 3 (Fig. 4b), and was greatly reduced in the majority of tumours of grade 1.

CLDN4 was absent or its expression was greatly reduced in tumour cells of 17 out of 21 grade 1 tumours (Fig. 4c), but it was present in the normal epithelial components within the same blocks (14 IDC NST [no special type], 5 special types, 2 ILC). Positivity was seen in 8 out of 11 grade 2 tumours and in 5 out of 7 grade 3 invasive carcinomas. In the normal tissue surrounding grade 2 and 3 tumours, the CLDNs were consistently present. CLDN4 was absent or its expression was decreased in the seven special-type breast carcinomas (mucinous, tubular, papillary) but it was present in the normal tissue surrounding these tumours. In contrast to CLDN1, CLDN4 was consistently absent in regions of apocrine metaplasia (Fig. 4d).

### Real time-PCR results

Real-time PCR using CLDN1, CLDN3, CLDN4 and GAPDH specific primers was performed in the 17 samples detailed above. mRNA was expressed in all of the analyzed cases. Relative quantification using GAPDH as the reference gene was applied [20]. mRNA expression in 13 invasive tumours out of the 17 samples was analyzed as the sample group, and the surrounding breast tissue was used as the control group. During the real-time PCR reaction, CLDN1 mRNA was detected in 12 out of 13 analyzed tumours and in 12 out of 13 corresponding surrounding nontumourous breast tissue samples. In normal, surrounding breast tissue, the mean value of crossing points (cycle threshold) was 34,02 whereas in the tumour tissue samples it was 36,22 (indicating low levels of CLDN1 mRNA). CLDN1 was downregulated by 12-fold in the sample (tumour) group in comparison with the control group using GAPDH as the reference gene. CLDN3 mRNA was present in all tumours and surrounding tissues. CLDN3 exhibited no expressional difference between invasive tumours and nontumourous tissue samples.

CLDN4 mRNA was also found to be present in these 13 invasive tumours and surrounding mammary tissues using real time-PCR. According to relative quantification, CLDN4 mRNA expression did not appear to be changed in the invasive tumours in comparison with the surrounding normal tissue. It is important to emphasize that there were only two grade 1 invasive tumour samples among the 13 investigated invasive tumours.

The program used by us and described by Pfaffl and coworkers [20] (Relative Expression Software Tool [REST] for group-wise comparison and statistical analysis of relative expression results in real-time PCR) includes a statistical analysis (analysis of variance). Using this program, we found no statistically significant differences in the distribution of data between the sample and control groups in terms of CLDN expression (the *P *values for CLDN1, CLDN3 and CLDN 4 were 0.166, 0.928 and 0.996, respectively). However, only 13 tumours were analyzed for RNA expression using real-time PCR because fresh tumour tissue and corresponding normal tissue surrounding the tumour were available in only 13 cases. In these cases the results of immunohistochemistry and real-time PCR appeared to correlate; specifically, CLDN1 appeared to be downregulated in tumours as compared with normal breast, whereas CLDN3 was expressed at similar levels in tumours and normal breast.

In the case of CLDN4 expression the differences observed by immunohistochemistry between tumours of different grade were not observed by real-time PCR. CLDN4 mRNA expression did not appear to be changed in the invasive tumours in comparison with the surrounding normal tissue. Real-time PCR remains to be performed in a greater number of cases, including tumours of different grade.

Apart from melting analysis, the specificity of real-time PCR results was confirmed on 2% agarose gel electrophoresis. The PCR product was seen at the appropriate size and nonspecific bands were not visualized. The specificity of real-time PCR of CLDN4 was also analyzed by sequencing (data not shown).

## Discussion

In the present study we examined the expression in breast lesions of three members of the CLDN family, namely CLDN1, CLDN3 and CLDN4. The majority of studies published to date have described roles for CLDNs in forming TJs [22], conferring ionic selectivity [17] and functioning as a barrier [23], but only few data have been published on the expression of CLDNs in the breast [14]. CLDN1 and CLDN2 were the first described CLDN components of TJs, and so the majority of data are related to these proteins. It was demonstrated that CLDN1 and CLDN2 function as major structural components of TJ strands, and that occludin is an accessory protein within this structure [8,24].

Well developed TJs found in the lung contain CLDN3, CLDN4 and CLDN5, which are abundant, whereas levels of expression of CLDN1 and CLDN2 are reduced [12]. These findings suggest that different members of the CLDN family are involved in the formation of TJ strands in different tissues. The extent to which CLDN expression is tissue dependent remains a subject of research interest, as does the physiological relevance of the existence of multiple CLDN species. In CLDN1-, CLDN2- and CLDN3-transfected mouse L-cells, Kubota and coworkers [25] showed that CLDNs may act as calcium-independent adhesion molecules, although the adhesion strength is low. It was hypothesized by Rangel and coworkers [26] that the precise ratio of different CLDNs determined the permeability of TJs. In ovarian tumours, the same group suggested that CLDN3 and CLDN4 are required for signalling through survival or proliferative pathways.

In the present study, immunohistochemistry revealed expression of CLDNs in TJs along the lateral plasma membranes of epithelial cells and along the basal plasma membrane of the epithelium, suggesting that CLDNs are not exclusively localized to TJs. Localization of these three CLDNs together in breast tissue sections has not previously been reported, but based on the observations presented here CLDN1, CLDN3 and CLDN4 are not exclusively localized to areas of TJs. Gregory and coworkers [27] described similar observations for CLDN1 in the rat epididymis.

We demonstrated that immunohistochemically detectable CLDN1 protein is absent or its expression is markedly decreased in the majority of different types of invasive breast carcinomas as compared with normal ducts. Of 39 malignant tumours examined by immunohistochemistry, 36 were negative for CLDN1 or it was present in a scattered distribution among the tumour cells. CLDN1 mRNA expression investigated by real-time PCR confirmed this finding. The mRNA level in tumour is considerably lower than that in normal breast tissue. Theoretically, several regulatory pathways could influence CLDN1 protein synthesis and expression. How these regulatory pathways are involved in cellular transformation and oncogenic progression in the breast remains to be elucidated. Previous studies reported that CLDN1 mRNA expression is absent or downregulated in the majority of breast cancer cell lines [28]. Kramer and coworkers [7] found no evidence for alterations in the CLDN1 gene being responsible for the loss of expression of CLDN1 protein. The same group found a similar loss of CLDN1 expression in five primary breast tumours. This finding is in contrast to the broad spectrum of CLDN1 expression found in other human tissues, such as liver and pancreas (our unpublished data). This may suggest that CLDN1 might be involved in the development of breast cancer or other epithelial tumours [7]. Whether TJs are simply lost in breast neoplasia and whether downregulation of CLDN1 is among the causative factors in this process are unknown. Alterations in the number, appearance and permeability of TJs have been described in various tumour types [21,29]. Abnormalities in TJ permeability and number greatly influence the transepithelial flux of growth factors [30] and hence the development of epithelial tumours [31]. In two cases, both normal breast tissue and tumour sample were investigated by transmission electron microscopy. Membrane densities corresponding to TJ structures were identified in both normal and neoplastic epithelial cells. The eventual abnormalities of TJs in breast tumours must be analyzed in a large series of breast tumours.

Although the similar CLDN3 and CLDN4 TJ proteins are highly expressed in breast neoplasia, we hypothesize that mechanisms other than alteration in TJs are involved in the loss of CLDN1 expression in breast tumours. The role played by CLDN1 positivity in apocrine cells observed in this study requires further investigation. In the present study, CLDN3 appeared to be expressed in a similar manner in primary breast cancers as in normal epithelium. Immunohistochemistry and real-time PCR yielded similar findings without notable upregulation or downregulation in different groups of tumours or compared with normal epithelium. In the majority of cases, CLDN3 was found to be localized along the entire membrane of epithelial cells, but in some cases it was only detected close to the apical end or at the basolateral membrane of the epithelial cells. Although this observation was confirmed by both light and confocal microscopy, in the breast this staining pattern has not previously been reported. Interestingly, Rangel and coworkers [26] did not find CLDN3 exclusively at the membrane in any of the samples studied; the majority of samples of primary ovarian tissue exhibited a combination of cytoplasmic and membrane staining. In gut, Rahner and coworkers [32] found CLDN3 to be strongly expressed in the surface epithelial cells of the stomach fundus, and have found it to be distributed predominantly along the basolateral membrane and not at the TJ. That group concluded that CLDNs have different patterns of expression in different gastrointestinal tissues – patterns that should account for differences in paracellular permeability.

We found that CLDN4 protein expression was downregulated in grade 1 IDCs. Further sample collection and analysis are required to confirm this result at the mRNA level, because only two grade 1 IDCs were available for PCR examination in our series. However, mRNA and protein were expressed in grade 2 and 3 IDCs to degrees similar to expression in surrounding breast tissue. In a study conducted in ovarian tissue, Hough and coworkers [16] found that expression of CLDN3 and CLDN4 protein was undetectable in normal ovarian cells and highly expressed on the membranes of ovarian carcinoma cells. Contrary to the findings in ovarian tumours reported by Rangel and coworkers [26] that CLDN3 and CLDN4 exhibited cytoplasmic staining and that over-expression of these proteins were associated with malignancy, in our study in breast we did not observe clear positivity of CLDNs in cytoplasm. A close relation between CLDN4 expression and changes in both conductance and ionic selectivity has been described. Further important observations were reported by van Itallie and coworkers [17]; they found that the number of junction strands was increased by over-expression of CLDN4, and that the levels of several other strand proteins were unaffected. Loss of CLDN4 from the cell surface coincides with dramatic changes in the morphology of TJ fibrils. Removal of CLDN4 disrupts fibril organization and increases junction permeability [15]. A recent study [33] identified CLDN4 as a potent inhibitor of the invasiveness and metastatic phenotype of pancreatic carcinoma cells by playing role in the transforming growth factor-β and Ras/Raf signal regulated kinase pathway. No studies to date have reported decreased expression of CLDN4 in grade 1 breast carcinomas, in special types of breast tumour, or in apocrine metaplasia compared with normal epithelium, as were observed in our study. The mRNA expression of different CLDNs in a large number of special-type breast carcinomas and tumours of different grades requires investigation so that the decrease in CLDN4 expression in special-type breast carcinomas and IDCs of grade 1 can be unequivocally confirmed.

## Conclusion

Our observations suggest that, in breast tissue, CLDN3 expression is similar in tumours and surrounding normal tissue, as demonstrated by immunohistochemistry and real-time PCR. In contrast, absent or decreased expression of CLDN1 in invasive breast carcinomas was demonstrated at both mRNA and protein levels. These data suggest that CLDN1 is involved in invasion and metastasis, and in mammary carcinogenesis. Furthermore, loss of or decrease in CLDN4 expression in grade 1 IDCs and the absence of CLDN4 in special-type breast carcinomas and in areas of apocrine metaplasia in benign breast tissue, as compared with normal epithelium, suggest a role for CLDN4 in cellular differentiation. The implications of this unique feature require further investigation. The important issue is whether loss of CLDN1 and CLDN4 plays a significant role in cell–cell adhesion and tumour differentiation.

## Abbreviations

CLDN = claudin; DCIS = ductal carcinoma *in situ*; GAPDH = glyceraldehyde-3-phosphate dehydrogenase; IDC = invasive ductal carcinoma; ILC = invasive lobular carcinoma; OD = optical density; PCR = polymerase chain reaction; TJ = tight junction; ZO = zonulae occludens.

## Competing interests

The author(s) declare that they have no competing interests.

## Authors' contributions

A-MT and JK selected the cases, performed the pathological analysis (mainly JK), evaluated the immunohistochemical slides, performed the RT-PCR reactions (mainly A-MT) and wrote the manuscript. SP participated in the confocal microscopic investigation of the immunofluorescence slides. ÁS carried out the immunohistochemical reactions. CP participated in the evaluation of the RT-PCR reactions, the statistical analysis of the data and the writing of the manuscript. PKN participated in designing the CLDN primers. LS was the designer of the CLDN primers. AK participated in the evaluation of the RT-PCR reactions and the statistical analysis of the data and in the writing of the manuscript. KB participated in the evaluation of the immunohistochemical reactions and in the writing of the manuscript. ZS participated in the evaluation of the immunohistochemical reactions and in the writing of the manuscript in its final stage.

## Figures and Tables

**Table 1:** The CLDN1, CLDN3, CLDN4 and GAPDH primer sequences, position and product size

Primer	Forward			Reverse			Product size (bp)	GI number
			
	Sequence 5'–3'	Size (bp)		Sequence 5'–3'	Size (bp)			
CLDN1	ttcgtacctggcattgactgg	378–399	22	ttc gta cct ggc att gac tgg	470–490	21	112	4559277
CLDN3	ctgctctgctgctcgtgtcc	732–751	20	ttagacgtagtccttgcggtcgtag	836–860	25	128	21536298
CLDN4	ggctgctttgctgcaactgtc	741–761	21	gagccgtggcaccttacacg	829–848	20	107	14790131
GAPDH	gaagatggtgatgggatttc	81–98	20	gaaggtgaaggtcggagt	287–306	18	225	7669491

bp, base pairs; CLDN, claudin; GAPDH, glyceraldehyde-3-phosphate dehydrogenase.
